# Study on the characteristics of nitrogen dioxide adsorption and storage of coal residue in coal-fired power plants in goaf

**DOI:** 10.1038/s41598-021-87855-y

**Published:** 2021-04-23

**Authors:** Xuefeng Wang, Ling Qiao, Cunbao Deng, Ge Chu, Xiaofeng Li, Qi Zhao, Guoli Wang

**Affiliations:** 1 College of Safety and Emergency Management Engineering, Taiyuan University of Technology, Taiyuan, 030024 China; 2 College of Safety Science & Engineering, Liaoning Technical University, Fuxin, 123000 China; 3 China Coal Society, Beijing, 100000 China; 4 Dananhu No.1 Mine of Guoshen Group, Hami, 839000 China

**Keywords:** Environmental sciences, Chemistry, Energy science and technology

## Abstract

In order to realize the storage of the residual coal in the goaf on the flue gas of the power plant, the adsorption characteristics of nitrogen dioxide in the flue gas of the power plant were studied. The Gaussian09 was used to study the adsorption process of NO_2_ molecules on coal at the density functional (DFT) B3LYP/6-311G level, and the model of NO_2_ adsorption by coal was established. Different quantities were obtained using orbital energy changes and molecular bond length changes. According to the principle of molecular adsorption, the adsorption of NO_2_ by coal is considered to be physical adsorption with endothermic heat. On the basis of simulation, using self-organized experimental devices, the single-component NO_2_ gas and the simulated coal-fired power plant flue gas were introduced into anthracite, bituminous coal and lignite. In single-component adsorption, the adsorption of NO_2_ by lignite increases with time. The time to reach equilibrium is related to the properties of the coal itself. In the process of simulated flue gas adsorption, the order of the adsorption amount of coal to flue gas is CO_2_ > NO_2_ > N_2_ > O_2_. In the simulated flue gas, coal is easy to absorb NO_2_ and CO_2_, and the competition between gases reduces the frequency of contact between NO_2_ and the coal surface. Simulation and experimental results show that coal has obvious adsorption characteristics for NO_2_, and it is feasible for the residual coal in the goaf to adsorb NO_2_ in the flue gas of power plants.

## Introduction

The presence of nitrogen oxides in the flue gas is an important gas that causes the ozone layer, and particle pollution and related pollution are also the main cause of acid rain, which will destroy the ozone layer. The nitrogen oxides in human production and life mainly come from the burning of fossil fuels, and the content in the air increases year by year. The nitrogen oxide content in the flue gas of coal-fired power plants is about 0.05%. According to relevant reports and studies, the amount of NO_X_ emissions from fuel combustion in thermal power plants, iron smelters, and chemical plants accounts for more than 90% of total man-made emissions^1–4^. In order to deal with NO_X_ in the flue gas of power plants, dry flue gas denitration and wet flue gas denitration are often used to reduce NO_X_ in flue gas. In the dry flue gas denitration, the SCR catalyst is difficult to choose and the cost is high; the SNCR denitration rate is low and the temperature is difficult to control. The NH_3_ and flue gas produced by the combined denitrification technology of the two methods in the SNCR area cannot meet the denitrification demand of the SCR area. The catalytic decomposition method has a low denitration rate. The adsorption method is susceptible to interference from external conditions and has high requirements for NO_X_. Wet flue gas denitration technologies include reduction adsorption, oxidation adsorption and lye absorption. The use of lye adsorption is limited because of its high cost and complex products. The oxidation absorption method requires the equipment to have anti-corrosion ability, and the conversion material is still polluting the environment. Although the reduction and absorption method is harmless, the denitrification rate is low and cannot meet the atmospheric emission standards. The above-mentioned methods have serious disadvantages and cannot be promoted vigorously. Therefore, the denitration of flue gas in power plants is still an urgent problem to be solved^5–10^. In the 1970s, the United States took the lead in researching coal-bed methane as a resource and achieved success. Later, the research on coal adsorbed gas gradually deepened. Experiments show that there are many types of coal adsorbed gases, including CH_4_, N_2_, CO_2_, O_2_, H_2_, and NO_2_^12^.

Coal adsorbs gas through van der Waals force, and the van der Waals force is related to the properties of the coal itself. In recent years, the research on the affinity of coal to various gases has achieved many remarkable results in the aspects of experiment and simulation calculation^13^.

The competitive adsorption and diffusion mechanism of CO_2_/CH_4_/H_2_O mixture in lignite was studied by using Monte Carlo method and molecular dynamics. The effects of temperature and pressure on competitive adsorption and diffusion behavior were discussed. The results show that the adsorption capacity of lignite to CO_2_ is greater than that of CH_4_, and the adsorption of moisture on gas is inhibited. Xu et al. conducted a grand canonical Monte Carlo simulation on the bituminous coal model, studied the adsorption behavior of CO_2_, CH_4_ and their mixtures, and believed that CO_2_ showed preferential adsorption over CH_4_. Li et al. established 3 models of different coal ranks and studied the competitive adsorption of CO_2_/CH_4_ on coal. The results show that from low-rank coal to high-rank coal, the total pore volume, porosity and effective pore ratio of coal increase, which leads to an increase in the adsorption capacity of coal. With the increase of coal rank, the choice of CO_2_/CH_4_ adsorption Sexual decrease. Yang et al. established a gas–solid coupling test system and performed uniaxial loading tests on coal samples adsorbed with different gases, indicating that under the same adsorption pressure, the adsorption capacity and degradation rate of coal for CO_2_, CH_4_, N_2_ and He decreased in turn. Kui et al. proposed that when the coal body expands with the increase of water content, the adsorption capacity and adsorption rate of CH_4_ both decrease. Lin et al. studied the adsorption behavior of bituminous coal in mixed gases with different ratios, and established a coal adsorption experimental system to conduct experiments. The results showed that the adsorption of bituminous coal to CO_2_ is stronger than that of N_2_, and there is a competitive adsorption relationship between the two. Stevenson et al. used dry coal samples to perform adsorption tests on CH_4_, N_2_, and CO_2_ mixed gases. Arri and Yee used wet coal samples to study the adsorption experiment of CH_4_, CO_2_ and CH_4_, N_2_, and obtained that the adsorption of coal to multiple gases was achieved by competing for the same adsorption sites. Cui et al. studied the isothermal adsorption experiment of binary mixed gas of CH_4_, CO_2_, N_2_ with different ratio concentrations, and obtained the different adsorption capacity of each component gas under different conditions. Gu et al. simulated the adsorption process of coalbed methane (CH_4_, N_2_), and the quantitative relationship between the concentration of the component in the free phase and its concentration in the adsorbed phase was determined by the adsorption competition. Yu et al. studied the adsorption characteristics of CH_4_ and CO_2_ and their mixed gas under high pressure, and found that the adsorption capacity of coal for the mixed gas is between that of pure CH_4_ and pure CO_2_. Wang et al. applied quantitative calculations to study the mixed adsorption process of multiple gases on the surface of coal, and concluded that the affinity order of adsorption on the surface of coal with various gases is: O_2_ > H_2_O > CO_2_ > N_2_ > CO > CH_4_. However, most of the current researches focus on the competitive adsorption between CO_2_, N_2_ and coalbed methane, and there are few studies on the adsorption of NO_2_.

The adsorption of coal and gas is not only on the surface, but also on the surface of the pores in the coal. In this study, the simplified structure of the coal surface and the physical adsorption of gas were selected. However, the adsorption of gas in coal pores also includes physical adsorption, associative chemical adsorption and dissociative chemical adsorption. In addition, the adsorption may cause deformation of the pores, and the phenomenon of pore blockage, which leads to a decrease in pore volume, will affect the adsorption. This paper only studies the physical adsorption process of coal and gas.

## Gaussian simulated coal adsorption of NO_2_

The calculation was completed with the Gaussian09 program. The adsorption process of coal molecules and NO_2_ molecules was studied at the level of density functional (DFT)B3LYP/6-311G.

### Simplify model building

**Figure 1:**
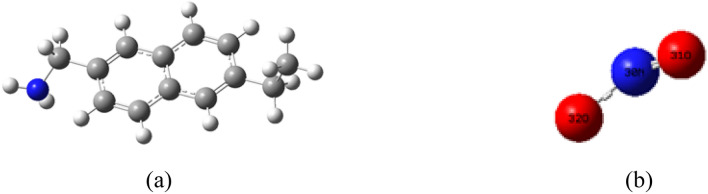
Geometrically balanced configuration of coal surface (**a**) and NO_2_ molecule (**b**).

It is generally believed that coal is a polymer structure, which is based on condensed aromatic ring and connected with various alkyl side chains, oxygen-containing functional groups and bridge bonds. The composition of coal is also relatively complex, mainly composed of carbon, hydrogen, oxygen, nitrogen and sulfur, and many kinds of trace elements. The composition and structure of coal vary greatly according to its metamorphic degree and geological conditions. In the simulation calculation, the more complex the coal structure model, the less representative it is. In the artical, the model of molecular fragments on the surface of coal is simplified as a characteristic structure of coal composed of two benzene rings. A side chain containing 1 C atom, 1 N atom and a side chain containing 2 C atoms extend from the skeleton. The model removes the three-dimensional layered structure of coal molecules, and retains the benzene ring and side chain structure. Geometrically balanced configuration of coal surface and NO_2_ molecule is shown in Fig. 1.

### Calculation results

For the adsorption of single and multi-molecule NO_2_ molecules on the coal surface, the frontier orbital energy of the optimized physical adsorption structure is shown in Table 1.

**Table 1:** Orbital energy of each state front.

Molecule	HOMO (ev)	LUMO (ev)
NO_2_	− 0.12774	− 0.30700
C_13_H_15_S	− 0.21366	− 0.03672

It can be seen that the highest occupied orbital energy on the coal surface is higher than the highest occupied orbital energy for NO_2_, and the difference between the highest occupied orbital energy of coal and the lowest empty orbital energy of NO_2_ molecules is relatively small. It shows that the adsorption of NO_2_ molecules on the surface of coal is that the surface of coal provides electrons for NO_2_ molecules.

**Figure 2:**
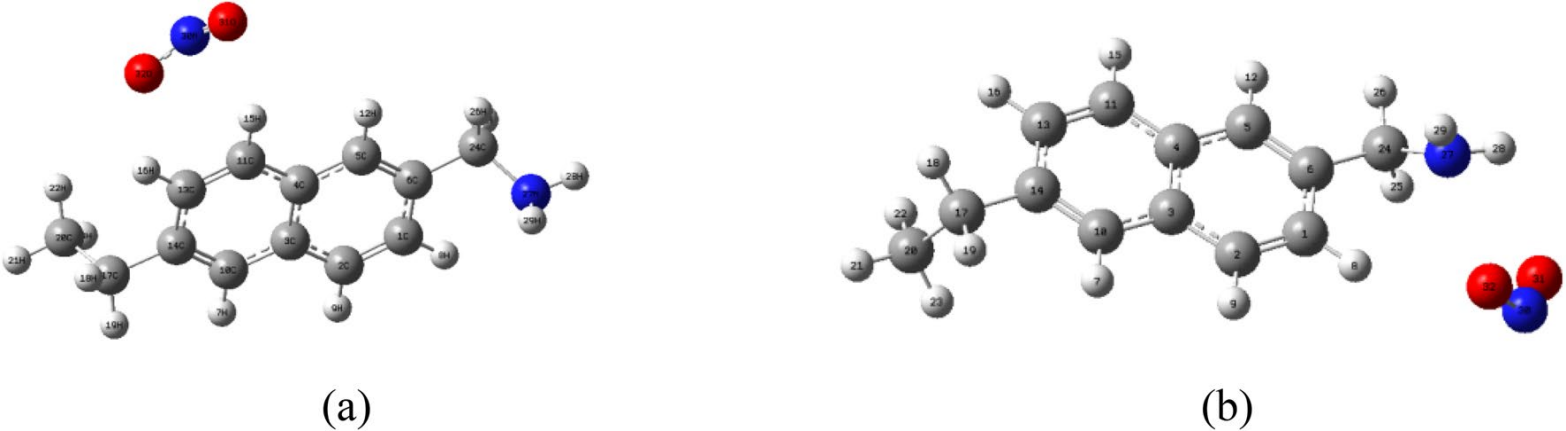
Equilibrium configuration diagram of single NO_2_ molecule adsorption on benzene ring (**a**) and side chain (**b**) on coal surface.

As shown in Fig. 2a, when a single NO_2_ molecule was adsorbed on the benzene ring on the coal surface, the bond length of the NO_2_ molecule changes from R (30,31) 1.2376 Å before adsorption and R (30,32) 1.2376 Å to after adsorption R(30,31) 1.23359 Å, R(30,32) 1.23611 Å. As shown in Fig. 2b, the bond length of CN of the side chain on the coal surface changed from 1.46731 to 1.46555 Å; the bond length of the NO_2_ molecule changed from R (30,31) 1.2376 Å before adsorption and R (30,32) 1.2376 Å to adsorption After R(30,31) 1.242 Å and R(30,32) 1.2413 Å. It can be seen that the C–N bond length of NO_2_ molecules has little change, and the bond length of NO_2_ molecules is obviously elongated, and the change is greater than that of single NO_2_ molecule adsorption on the benzene ring on the coal surface. This indicates that the adsorption of a single NO_2_ molecule on the coal surface side chain contributes more to the adsorption of NO_2_ molecules on the coal than on the benzene ring. Therefore, the side chain groups on the coal surface are more likely to physically adsorb with NO_2_ molecules.

**Figure 3:**
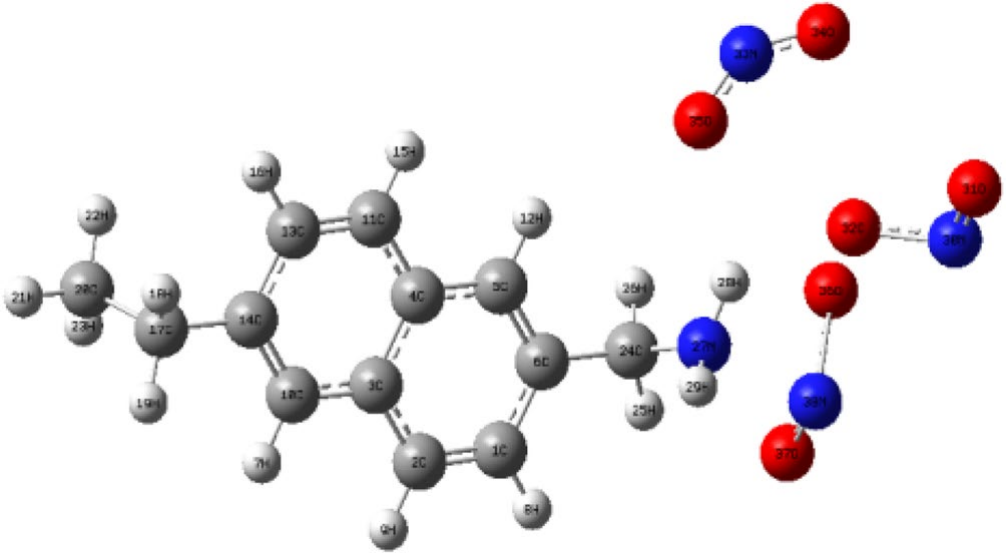
Adsorption equilibrium configuration of three NO_2_ molecules on the coal surface side chain.

As shown in Fig. 3, Tables 2 and 3, for the NO_2_ molecules adsorbed on the side chains on the coal surface, the bond length of the NO_2_ molecules changed from 1.2376 Å before adsorption to 1.5828 Å, 1.6369 Å and 1.7905 Å. The calculation results show that the bond lengths of the three NO_2_ molecules adsorbed on the side bonds are all elongated, which increases the activity of the NO_2_ molecules and makes them more likely to react with the coal surface. When the C-N bond is on the side chain of coal adsorbs NO_2_ molecules, the bond length changes from 1.4673 to 1.4661 Å. It can be seen that although the bond length of the C-N bond does not change much, the bond length of the C-N bond becomes shorter as the number of adsorbed NO_2_ molecules increases. Figure 3 is the adsorption equilibrium configuration diagram of the side chains of three NO_2_ molecules on the coal surface. Comparing the changes in the surface structure of the coal, it is found that the bond length and bond angle on the coal surface do not change much after adsorption. This also proves that the three NO_2_ molecules are in the adsorption on the coal surface is physical adsorption. Comparison of infrared spectrum frequency before and after adsorption of three NO_2_ molecules and coal surface side chain is shown in Table 4.

**Table 2:** Bond lengths of three NO_2_ molecules on the coal surface side chain adsorption equilibrium configuration.

Atomic relationship	Bond length (Å)	Atomic relationship	Bond length (Å)	Atomic relationship	Bond length (Å)
R(8,32)	2.9711	R(24,27)	1.4661	R(27,32)	2.7214
R(28,30)	2.3521	R(28,31)	2.2218	R(28,32)	3.076
R(28,35)	2.7556	R(30,31)	1.5828	R(30,32)	1.1917
R(31,33)	2.4532	R(31,35)	1.4311	R(33,34)	1.1903
R(33,35)	1.6369	R(38,36)	1.7905

**Table 3:** Bonding angles of three NO_2_ molecules on coal surface side chains.

Atomic relationship	Key angle (angle)	Atomic relationship	Key angle (angle)	Atomic relationship	Key angle (angle)
A(1,8,32)	129.6643	A(24,27,32)	82.2518	A(29,27,32)	118.1455
A(27,28,30)	65.9684	A(27,28,31)	111.6387	A(27,28,35)	110.0297
A(30,28,35)	60.5597	A(31,28,32)	52.7023	A(32,28,35)	53.582
A(31,30,32)	109.0978	A(28,31,33)	129.1057	A(30,31,33)	98.1965
A(30,31,35)	105.9303	A(8,32,27)	69.0264	A(8,32,28)	87.7281
A(8,32,30)	100.1664	A(27,32,30)	49.5486	A(31,33,34)	142.733
A(34,33,35)	109.0376	A(28,35,33)	150.6746

**Table 4:** Comparison of infrared spectrum frequency before and after adsorption of three NO_2_ molecules and coal surface side chain (cm^−1^).

Name	Before adsorption	After adsorption	Name	Before adsorption	After adsorption
${\upnu}$ C3-C4	1411.31	1415.67	${\upnu}$ C11-C13	1649.38	1648.44
${\upnu}$ C1-C6	1606.68	1604.82	${\upnu}$ C10-C14	1649.38	1648.44
${\upnu}$ C13-C14	1606.68	1604.82	${\upnu}$ C4-C5	1674.44	1673.94
${\upnu}$C5-C6	1649.38	1648.44	${\upnu}$C4-C11	1674.44	1673.94
${\upnu}$C3-C10	1674.44	1673.94	${\upnu}$C24-H26	2928.20	2969.09
${\upnu}$C24-H25	3012.99	3105.57	${\upnu}$C5-H12	3142.73	3153.84
${\upnu}$N24-H25	3517.59	3506.05	${\upnu}$N30-O31	1252.72	1249.97
${\upnu}$N33-O34	1252.72	1249.97	${\upnu}$N38-O36	1252.72	1249.97

The calculation formula of adsorption energy is:

1 $${\text{E}}_{{{\text{ads}}}} = {\text{E}}_{{\text{c}}} + {\text{E}}_{{{\text{NO}}_{2} }} - {\text{E}}_{{{{{\text{NO}}_{2} } \mathord{\left/ {\vphantom {{{\text{NO}}_{2} } {\text{C}}}} \right. \kern-\nulldelimiterspace} {\text{C}}}}}$$

Among them: *E*_*ads*_—the adsorption energy of coal surface and oxygen molecules reaches the equilibrium adsorption energy; *E*_*C*_—the energy before adsorption occurs on the coal surface; $E_{{{\text{NO}}_{2} }}$—the energy before adsorption of NO_2_ molecules; $E_{{{{{\text{NO}}_{2} } \mathord{\left/ {\vphantom {{{\text{NO}}_{2} } {\text{C}}}} \right. \kern-\nulldelimiterspace} {\text{C}}}}}$—the total energy of the entire adsorption system after NO_2_ molecules are adsorbed on the coal surface.

The energy of the NO_2_ molecule before adsorption is − 205.05196016 Hartee, the energy of the coal surface is − 559.16847497 Hartee, and the energy of the adsorption state composed of three NO_2_ molecules and the side chain on the coal surface is − 1174.31977016 Hartee. According to the formula (1), the adsorption energy composed of three NO_2_ molecules and coal surface is 12.04 kJ/mol. This shows that the process of coal molecules adsorbing three NO_2_ molecules is an endothermic process.

## Experimental study

### Experimental device

**Figure 4:**
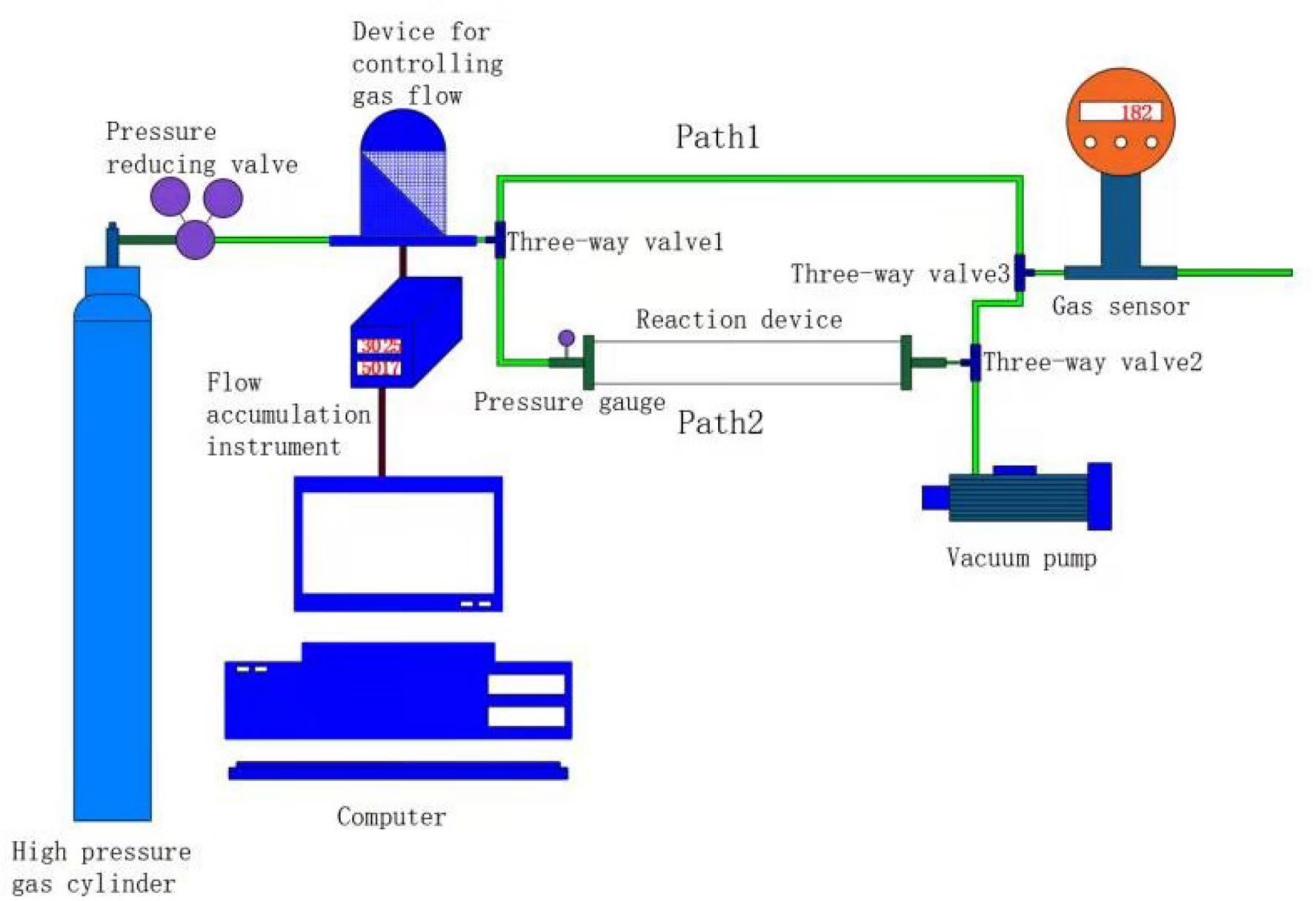
Experimental device diagram.

This experiment was carried out at room temperature and pressure, as shown in Fig. 4, the device includes: (a) high-pressure gas cylinder; (b) Pressure reducing valve, buffer gas released from high-pressure gas cylinder; (c) Gas flow meter and totalizer; (d) Pressure gauge; (e) The reactor uses a quartz tube with an outer diameter of 30 mm, a wall thickness of 3 mm, and a length of 300 mm, and the two ends are vacuum-sealed; (f) NO_2_ gas sensor (when simulating flue gas, smoke Four sensors of gas composition are connected in series); (g) Vacuum pump, the reactor is evacuated to a vacuum state before the experiment. (h) Computer, control flow meter. The device can simulate the temperature and pressure of the goaf, and the experimental data provide a basis for exploring the CO_2_ storage volume in the goaf and whether NO_2_ can be stored under the premise of CO_2_ saturated adsorption.

### Experiment

Sample preparation: The first step was to crush the coal sample. Put the coal sample into a ball mill for crushing, then sieving, put the coal sample smaller than 50 mesh into a reagent bottle, label it, and then put it in a vacuum drying box for storage.

Experimental operation: (1) Add the sample to be tested before the experiment starts, close valve 1 and turn valve 2 to the direction of vacuum pump, use the vacuum pump to evacuate the reactor for three hours. (2) Install the software independently developed by Qixing Company on the computer, and pass the gas flow Meter and totalizer to control the gas flow out of the high-pressure gas cylinder, set 50 ml/min, turn the valve 1 to the path 1, and the valve 2 to the path 1, after the gas comes out of the flowmeter, it passes through the valve 1, the path 1, the valve 3 Go directly to the gas sensor, calibrate the gas concentration of the gas cylinder, and compare it with the experimental data afterwards to get the true adsorption value. According to the value displayed by the sensor, calculate the real-time adsorption of NO_2_. Because coal does not adsorb argon, argon is used as the calculation standard. The calculation formula is as follows:

One-component NO_2_ experiment:

2 $${\text{V}}_{{{\text{NO}}_{2} }} = {\text{V}}_{{{\text{sum}}}} - \frac{{{\text{V}}_{{{\text{Ar}}}} }}{{1 - \varphi_{{{\text{NO}}_{2} }} }}$$

Simulated smoke:

3 $${\text{V}}_{{{\text{NO}}_{2} }} = {\text{V}}_{{{\text{Initial}}\;{\text{NO}}_{2} {\text{ volume}}}} - \frac{{{\text{V}}_{{{\text{Ar}}}} \varphi_{{{\text{NO}}_{2} }} }}{{1 - \varphi_{{{\text{CO}}_{2} }} - \varphi_{{{\text{NO}}_{2} }} - \varphi_{{{\text{N}}_{2} }} - \varphi_{{{\text{O}}_{2} }} }}$$

In the formula: ${\text{V}}_{{{\text{NO}}_{2} }}$ represents the real-time adsorption amount of NO_2_ gas; ${\text{V}}_{{{\text{Initial}}\;{\text{NO}}_{2} {\text{ volume}}}}$ represents the volume of CO_2_ coming out of the cylinder 8 ml/min; ${\text{V}}_{{{\text{sum}}}}$ represents the total volume of 50 ml/min coming out of the cylinder in one minute; ${\text{V}}_{{{\text{Ar}}}}$ represents the volume of argon for one minute, in different experiments; $\varphi_{{{\text{CO}}_{2} }}$ represents the value displayed by the CO_2_ gas sensor; $\varphi_{{{\text{NO}}_{{2}} }}$ represents the value displayed by the NO_2_ gas sensor; $\varphi_{{{\text{O}}_{{2}} }}$ represents the value displayed by the O_2_ gas sensor; $\varphi_{{{\text{N}}_{{2}} }}$ represents the value displayed by the N_2_ gas sensor.

(3) After three hours of evacuation, first close valve 2 and turn valve 1 to the direction of path 2. After the pressure gauge of the reactor shows atmospheric pressure, turn valve 2 and valve 3 to the direction of the gas sensor at the same time. (4) The sensor in the experimental device is real-time monitoring, and it takes a certain time to reach relative stability. Since the adsorption value changes greatly at the beginning of the experiment, the data is recorded every 10 min. The recorded data is the average value of the maximum value and the minimum value of the observation data within one minute as the recorded data. When the data change is small, the interval time can be appropriately increased for recording. (5) It is calculated that the amount of accumulated adsorbed gas in coal samples changes with time under different atmospheres.

### Results and discussion

#### Coal adsorption of single-component NO_2_

The ratio of gas used is 2% NO_2_ and 98% argon. The accuracy of the NO_2_ sensor is 0.0001%, and the condition is normal temperature and pressure. The experimental data of the cumulative adsorption of NO_2_ per gram of coal sample over time are shown in Table 5.

**Table 5:** Adsorption capacity of coal adsorbed single component NO_2_ gas with time.

Time(h)	Anthracite (ml)	Bituminous coal (ml)	Lignite (ml)
1	0.41387	0.43547	0.4177
2	0.6216	0.61637	0.5956
3	0.70036	0.68519	0.66442
4	0.73471	0.71464	0.69448
5	0.74461	0.72409	0.70369
6	0.75055	0.72877	0.70585
7	0.75505	0.73171	0.70765
8	0.75775	0.73321	0.70909
9	0.75931	0.73387	0.71017
10	0.76021	0.73447	0.71101
11	0.76057	0.73513	0.71179
12	0.76075	0.73555	0.71245
13	0.76105	0.73585	0.71275
14	0.76129	0.73603	0.71281
15	0.76135	0.73603	0.71287

**Figure 5:**
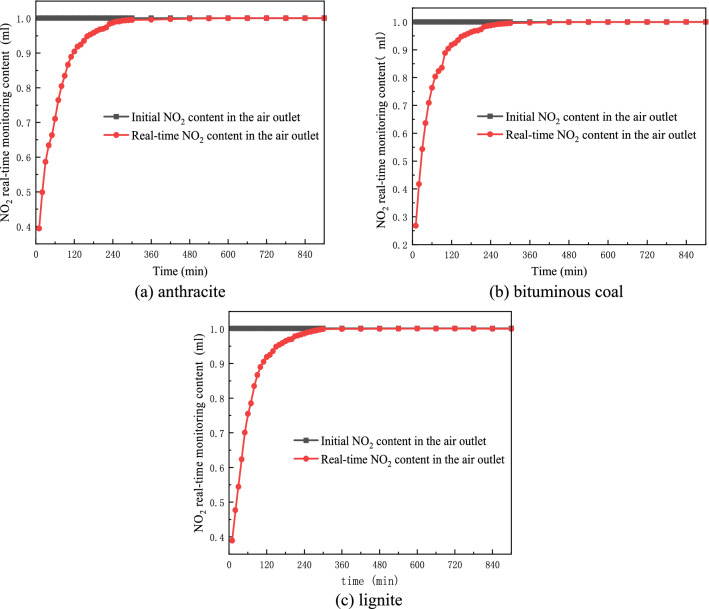
Change chart of NO_2_ content at coal sample outlet with time.

**Figure 6:**
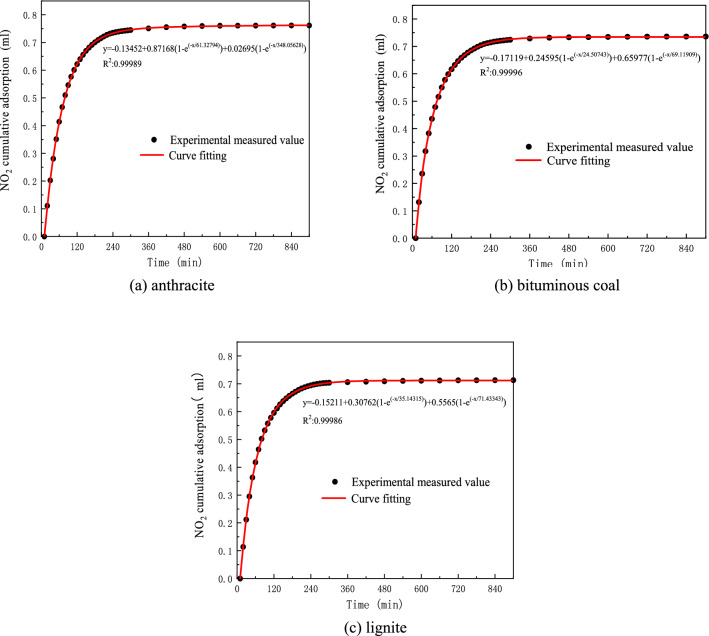
Cumulative adsorption of NO_2_ per gram of coal sample with time.

From the experimental data in Table 4 and Figs. 5, 6, it can be seen that lignite, bituminous coal, and anthracite have similar changes in the amount of NO_2_ adsorption, and they all accumulate over time. By analyzing the changes in the NO_2_ content of the gas outlets of the three coal samples, it was found that at the beginning of adsorption, the difference between the real-time content of the gas outlet and the initial content was the largest, indicating that the adsorption capacity was relatively large at the beginning of the experiment. With the passage of time, the difference between the real-time content and the initial content of the air outlet continues to approach 0, and the NO_2_ desorption adsorption reaches equilibrium. After 7 h, the adsorption amount of anthracite changed slowly, and the NO_2_ content at the outlet was basically the same as the initial content, which can be considered as saturated. After 7 h, the adsorption capacity of bituminous coal changes slowly, and it can be considered that the adsorption has reached saturation. The lignite reached the equilibrium state of analytical adsorption after 9 h.

#### Adsorption of simulated flue gas by coal

The adsorption of coal to simulated flue gas was based on the flue gas composition of the power plant, with a volume fraction of 79% N_2_, 16% CO_2_, 4.5% O_2_, 0.2% NO_2_, and the rest is argon. Three types of lignite, bituminous coal, and anthracite are used for the adsorption test of coal to the mixed gas. The conditions are set to normal temperature and pressure. The experimental data of the cumulative adsorption of four gases per gram of coal sample over time are shown in Table 6, 7, 8.

**Table 6:** Change of accumulated adsorption capacity of 1 g anthracite with time.

Time (h)	NO_2_ (ml)	CO_2_ (ml)	N_2_ (ml)	O_2_ (ml)
1	0.01048	0.7552	0.0077	0.01493
2	0.02216	0.8917	0.0144	0.02061
3	0.03075	0.9390	0.0194	0.02274
4	0.03615	0.9557	0.0224	0.02410
5	0.04011	0.9665	0.0245	0.02515
6	0.04287	0.9743	0.0263	0.02593
7	0.04533	0.9809	0.0269	0.02647
8	0.04737	0.9851	0.0275	0.02701
9	0.04869	0.9881	0.0281	0.02749
10	0.04935	0.9905	0.0287	0.02779
11	0.04971	0.9917	0.0293	0.02809
12	0.04989	0.9917	0.0293	0.02839

**Table 7:** Change of accumulated adsorption capacity of 1 g bituminous with time.

Time (h)	NO_2_ (ml)	CO_2_ (ml)	N_2_ (ml)	O_2_ (ml)
1	0.0103	0.7406	0.0090	0.01516
2	0.02201	0.8727	0.0165	0.02082
3	0.03066	0.9200	0.0215	0.02283
4	0.03603	0.9367	0.0245	0.02419
5	0.04029	0.9475	0.0263	0.02524
6	0.04383	0.9553	0.0275	0.02602
7	0.04689	0.9619	0.0281	0.02656
8	0.04959	0.9661	0.0287	0.0271
9	0.05169	0.9691	0.0293	0.02758
10	0.05301	0.9715	0.0299	0.02788
11	0.05361	0.9733	0.0305	0.02818
12	0.05379	0.9739	0.0305	0.02848

**Table 8:** Change of accumulated adsorption capacity of 1 g lignite with time.

Time (h)	NO_2_ (ml)	CO_2_ (ml)	N_2_ (ml)	O_2_ (ml)
1	0.0100	0.7214	0.0098	0.0148
2	0.0216	0.8541	0.0173	0.02043
3	0.03043	0.9014	0.0223	0.02251
4	0.03537	0.9181	0.0255	0.02387
5	0.03958	0.9289	0.0277	0.02492
6	0.04312	0.9367	0.0289	0.02570
7	0.04618	0.9433	0.0295	0.02624
8	0.04888	0.9475	0.0301	0.02678
9	0.05098	0.9505	0.0307	0.02732
10	0.05224	0.9529	0.0307	0.02768
11	0.05272	0.9547	0.0307	0.02798
12	0.05278	0.9553	0.0307	0.02828

**Figure 7:**
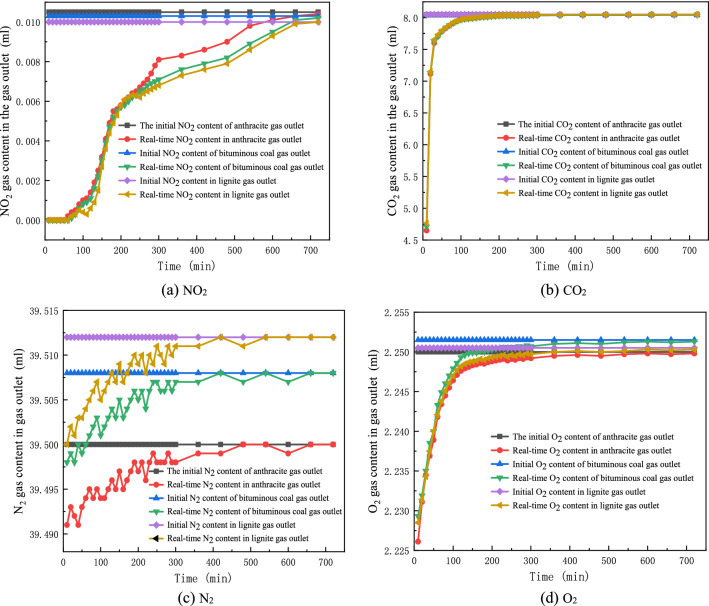
Change of gas content at the outlet of each coal sample with time.

According to the analysis of Table 5 and Fig. 7a, it is considered that the adsorption amount of NO_2_ by lignite, bituminous coal and anthracite is accumulated with the increase of time, and the increment gradually decreases in the later stage and reaches the equilibrium state. The adsorption capacity of anthracite, bituminous coal and lignite for NO_2_ at 12 h was 0.04989 ml/g, 0.05379 ml/g, and 0.05278 ml/g, respectively.

By comparing the experimental data of single-component and simulated flue gas, it is found that at the end of the experiment, under single-component conditions, the amount of NO_2_ adsorbed per gram of anthracite is 1.24144 g, the amount of NO_2_ adsorbed per gram of bituminous coal is 1.1894 g, and the adsorbed amount per gram of lignite It is 1.14454 g. The higher the degree of coal metamorphism is, the greater the saturated adsorption capacity of coal is. At the end of the simulated flue gas experiment, the amount of NO_2_ adsorbed per gram of anthracite was 0.05379 g, the amount of NO_2_ adsorbed per gram of bituminous coal was 0.05278 g, and the amount of adsorbed NO_2_ per gram of lignite was 0.04989 g. Compared with the single component, the saturated adsorption capacity is very different. The single component saturated adsorption capacity of anthracite is 23 times that of the simulated flue gas, the single component saturated adsorption capacity of bituminous coal is 22 times that of the simulated flue gas, and the single component saturated adsorption capacity of lignite The amount is 23 times that of the simulated flue gas. Except that the NO_2_ gas content is a factor that affects the saturated adsorption of NO_2_ by coal, the presence of other gases in the simulated flue gas compete with NO_2_ for adsorption.

Figure 7 is the curve of the four gas content changes with time of NO_2_, CO_2_, N_2_, O_2_ at the outlet of three coal samples. The experimental results show that the adsorption order of the four gases is as follows. The results show that the adsorption capacity of coal to flue gas is CO_2_ > NO_2_ > N_2_ > O_2_. Coal is a very complex organic mixture composed of many benzene rings and polar functional groups, which easily adsorb NO_2_ and CO_2_. The content of CO_2_ in the simulated gas is relatively high, so the competitive adsorption between CO_2_, NO_2_, N_2_ and O_2_ is weakened, and reduces the contact frequency between NO_2_ and coal surface.

## Conclusions

1. A model of molecular fragments on the surface of coal with retaining benzene ring and side chain structure is constructed. The NO_2_ molecules adsorbed on the side chains of the coal surface are calculated. The calculation results show that the bond lengths of the three NO_2_ molecules adsorbed by the side bonds of coal are elongated and larger. This increases the activity of NO_2_ molecules, making it easier to react with the coal surface. Comparing the changes of coal surface structure before and after adsorption, it shows that the three NO_2_ molecules are physically adsorbed.
2. When 2% NO_2_ mixed gas is introduced, anthracite, bituminous coal and lignite can store about 0.76m^3^/t of NO_2_, about 0.74m^3^/t of bituminous coal, and 0.71m^3^/t of lignite, respectively.
3. After introducing 0.2% NO_2_ simulated flue gas, anthracite can be stored at 0.05379m^3^/t, bituminous coal can be stored at 0.05278m^3^/t, and lignite can be stored at 0.04989m^3^/t. Due to competitive adsorption of NO_2_, CO_2_, N_2_, and O_2_ in power plant flue gas, there is an influence on the amount of NO_2_ adsorption.
4. Under normal temperature and pressure, the adsorption of NO_2_ by coal samples is physical adsorption, and the time to reach equilibrium is related to the properties of the coal itself.
